# Systemic lupus erythematous revealed by cytomegalovirus infection

**DOI:** 10.11604/pamj.2016.24.241.8988

**Published:** 2016-07-15

**Authors:** Rezgui Amel, Karmani Monia, Mzabi Anis, Ben Fredj Fatma, Laouani Chadia

**Affiliations:** 1 Internal Medicine Department, CHU Sahloul, Tunisia

**Keywords:** Systemic lupus erythematous, cerebral vasculitis, cytomegalovirus infection

## Abstract

Cytomegalovirus (CMV) infection have been described as exacerbing systemic lupus erythematous (SLE). The role of CMV in starting off SLE remains object of debate. We report a severe presentation of SLE revealed by CMV infection with hemophogocytic syndrome. A 22 old women without a history of systemic disease developed a cutaneous eruption with fever and myalgia persistant for 2 weeks. Laboratory studies revealed a CMV serology supporting acute CMV infection, with positive antinuclear antidody, anti ds DNA, elevated liver functions tests, pancytopenia. Further exams revealed an hemophagocytic syndrome and a lupus nephritis. While receiving antiviral and corticosteroid therapy, the patient developed seizures related to a cerebral vasculitis. The outcome was favorable when intravenous immunoglobulins were associated. This observation showed that CMV infection in patients with SLE is often serious and difficult to diagnose and to treat, especially when SLE is not yet recognized. So we suggest all patients with recent SLE have routine testing for CMV immunity.

## Introduction

The CMV infection in immunocompetent persons usually leads to acute hepatitis and mononucleosis infection, but when affects immunocompromised hosts, it may associate life threating and high mortality. The literature review suggest some evidence that CMV plays a role in inducing autoimmune responses such in the SLE [1, 2].

## Patient and observation

**Figure 1:**
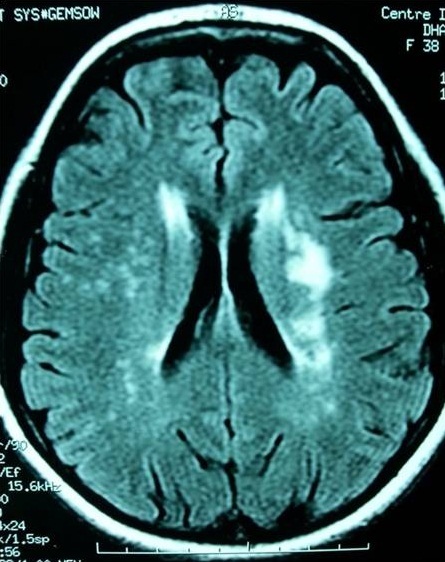
FLAIR axial MR image shows areas of hyperintensity within the subcortical white matter bilaterally, consistent with ischemic infarctions and suggestive of cerebral vasculitis

We present a 22 year old woman with no history of systemic disease, who developed a cutaneous eruption with arthromyalgia and fever persistant for two weeks. There was no infective endocarditis. The viral serologies showed elevated titers of Ig M antibodies to CMV, suggesting CMV infection. The CMV antigenemia test was also positive. In further laboratory studies, we found leucopenia (3000/µL), lymphopenia (800/µL), thrombocytopenia (110000/µL), hemolytic anemia, anti nuclear factor positivity with high titer of anti DNA (600 UI/ml). There was also proteinuria (4g/day) that indicated kidney biopsy. Histological examination revealed stage II lupus nephritis. The cutaneous biopsy showed a positive lupus band test. The bone marrow aspirate showed hemophagocytosis. Corticosteroids therapy was started with antiviral therapy (Ganciclovir). But the patient presented seizures and her cerebral magnetic resonance imaging showed images of cerebral vasculitis (Figure 1). Pulse cyclophosphamide therapy was indicated but the patient get worse with increasing titers of leucopenia, thrombocytopenia and severe cytolysis. So intravenous immunoglobulin were started and leaded to a favorable outcome. There was a slow normalization of liver tests, hemostasis parameters and urinary sediments without seizure recurrence.

## Discussion

A primary infection with CMV is usually asymptomatic but may associate mononucleosis syndrome. It often leads to immune dysfunction, especially an autoimmune phenomena [2]. Our report, such as others in the literature [3], showed that a severe CMV infection has revealed a LES with high activity disease. These findings raise the possibility that CMV infection may induce SLE in predisposed persons.

Mechanisms by which CMV can trigger autoimmunity have been proposed. In fact, it was proved that a C terminal peptide of CMV protein pp65 is highly immunogenic in patients with SLE and antibodies against this peptide cross react with nuclear proteins. These findings highlight the fact that immunization with one CMV peptide results in multiple auto reactive antibodies probably by molecular mimicry [2].

Our patient had presented a severe form of CMV infection with hemophagocytic syndrome. This entity is characterized by fever, pancytopenia, liver dysfunction and increased hemophgocytic histiocytes in the bone marrow, lymph nodes, liver and spleen [4]. Hemophagocytic syndrome is also associated with autoimmune diseases as like as SLE. Our case was considered to be induced by both CMV infection and SLE because of the high activity of the two diseases.

The occurrence of seizures in our report was explained by cerebral vasculitis finded at the MRI. CMV infection may be responsible of encephalitis but also cerebral vasculitis. Neurological involvement in SLE with cerebral vasculitis is an unusual entity. Indeed, large vessel vasculitis rarely involves the central nervous system (CNS) in patients SLE [5].

This diagnosis difficulty leads to a therapy challenge. Here in, Ganciclovir was early initiated with corticosteroids and hydroxylchloroquine. Cyclophosphamide was indicated for the CNS vasculitis but couldn’t be administrated because of the deep liver dysfunction. So we have opted for intravenous immunoglobulin. The early initiation of those therapies had improved our patient.

## Conclusion

Our case could support CMV infection as a potential trigger for SLE in predisposed persons. The clinical presentation may be so severe as it is illustrated with CNS vasculitis. Early initiation of treatment may improve the poor prognosis of such patients. Further studies can be interesting to establish suitable treatment for CMV-infection associated SLE. Patients recently diagnosed with SLE should have routine testing for CMV immunity.
